# NetZero deep brain stimulation: A carbon footprint minimisation audit and environmental impact analysis

**DOI:** 10.1016/j.bas.2026.106032

**Published:** 2026-04-07

**Authors:** Teresa Scott, Erlick AC Pereira, Michael G. Hart

**Affiliations:** Department of Psychology and Neuroscience, St George's School of Health and Medical Sciences, City St George's, University of London, Cranmer Terrace, London, SW17 0RE, UK

**Keywords:** Deep Brain Simulation (DBS), Neurosurgery, Neuromodulation, NetZero, Sustainability, Carbon footprint

## Abstract

**Introduction:**

The NHS accounts for around 5% of the UK's total carbon emissions, with surgical care contributing a significant proportion. We developed a “NetZero-DBS” workflow that we used to implement 5 high-impact changes to making surgery greener.

**Research question:**

To what extent can an optimised “NetZero-DBS” workflow reduce the environmental impact of DBS surgery through changes in waste management, consumable use, and product carbon footprint?

**Material and methods:**

A prospective audit compared sixteen consecutive DBS cases (eight before and eight after workflow implementation). Effectiveness was assessed through a structured waste audit, deep-dive product carbon footprint assessment, and consumable use appraisal.

**Results:**

The mean total waste generated per procedure before and after implementing our changes was 19 kg and 15.5 kg, respectively, but neither this nor individual waste categories were statistically significantly changed. The reusable DBS probe had the highest product carbon footprint, PCF (0.34 kgCO2e) compared to the single-use alternatives (0.117 kgCO2e and 0.145 kgCO2e). However, its environmental impact could be reduced by up to 56% per use when reused as intended and hence would make a net impact after three operations. Consumable use was reduced by 21.36kgCO2e or 12% of total per procedure.

**Discussion and conclusion:**

We provide a workflow that allows hospitals undertaking deep brain stimulation neurosurgery to minimise their carbon impact depending on individual features of their service. Implementation of our “NetZero-DBS workflow” is estimated to yield annual carbon savings of approximately 854.5 kgCO2e per year, equivalent to the emissions produced by driving 2176 miles in an average car.

## Highlights

• NetZero-DBS workflow cut carbon emissions by up to 12% per DBS procedure.
• Reusable DBS probe achieved net carbon benefit after three reuses.
• Established a multimodal framework to guide sustainable surgical decision-making.

## 1 Introduction

Climate change is one of the pre-eminent existential concerns of our times and is deeply connected to healthcare in terms of its impact on health, healthcare systems, and the way care is delivered. Ironically, healthcare is a major contributor to climate change, contributing approximately 5% to global emissions (Rodríguez‐Jiménez et al., 2023). In 2022, the NHS became the first healthcare organisation to embed emissions goals into legislation, with a key target being to reduce emissions by 80% between 2028 and 2032 (NHS England, 2022). Despite these laudable ambitions, research into how this is to be achieved in the real world is fledgling.

Surgery constitutes a major component of the NHS’ carbon footprint with electricity use and procurement of consumables being major carbon hotspots within operating theatres (Rizan et al., 2020). A variety of sage recommendations to making surgery greener have been proposed, most notable of which are the ‘Intercollegiate Green Theatre Checklist’ (Robb and Pegna, 2023) and the ‘The Green Surgery’ report produced in collaboration by the UK Health Alliance on Climate Change, the Centre for Sustainable Healthcare, and Brighton and Sussex Medical School (Brighton et al., 2023). A strength of these recommendations is their generalisability and ease of adoption, while also generating significant positive media attention. However, current checklists are by design generic, therefore many aspects of individual surgical procedures remain unaccounted. Developing more nuanced recommendations for specific surgical procedures will require consideration of the multiple factors involved in each decision during surgery, such as evidence of efficacy, financial implications, or logistical barriers.

**Fig. 1:**
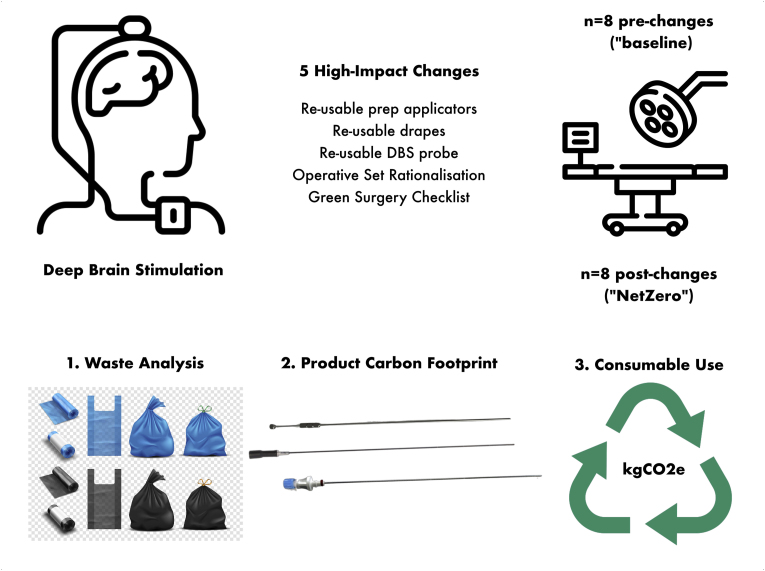
**Study Design** A prospective audit of 8 cases to document baseline waste use. We identified 5 high-impact changes that we could make to our DBS procedure (Table 1) then audited 8 consecutive cases subsequently. We assessed the effectiveness of our changes by weighing and categorising waste after each DBS procedure. Furthermore, we performed a high-level product carbon footprint (PCF) analysis of different probes used for creating a tract to place the DBS leads, as there was no good evidence of one over another and this was deemed to be the highest impact change within our audit. Finally, we performed a similar PCF analysis of the impact of items removed and added to the DBS procedure to estimate net reduction of kgCO2e per case.

Our aim was to develop a specific checklist for making deep brain stimulation (DBS) surgery greener (Fig. 1). This was chosen as it is a high-cost and potentially high-carbon device performed with moderate frequency in broadly uniform manner, yet with multiple permutations to each individual step. A key ambition was to deconstruct the procedure into its individual steps, which vary significantly between centres, and to provide guidance on how to weigh-up potentially more environmentally friendly options. The checklist was applied to identify five high-impact changes within our DBS surgery pathway. As previous studies have shown that a substantial proportion of waste generated in neurosurgical operating rooms derives from disposable items, we prioritised substituting disposable consumables with re-useable alternatives (Talibi et al., 2022; Zygourakis et al., 2017). Additionally, we included a deep-dive analysis of a single step in the pathway where multiple seemingly equivalent options were available. A prospective service evaluation before and after implementing this checklist was performed over 1 year in a single-surgeon's functional neurosurgery practice in the UK to evaluate the effectiveness of these recommendations, both in terms of waste generated and consumables used.

## 2 Methods

### 2.1 Ethics

Approval was provided by St George's NHS Foundation Trust as a Review of Service. Individual consent was not required.

### 2.2 Surgical technique

Our DBS workflow has been described previously (Mostofi et al., 2021). In essence, it comprises of MRI-planned CT-verified fully-asleep single-stage or staged surgery using a Cosman-Roberts-Wells (CRW) stereotactic frame with its ‘intubation’ head ring (HRAIM) and Brown-Roberts-Wells (BRW) localiser (IntegraLife, Princeton, New Jersey, USA). In this audit, only single-stage procedures were included.

### 2.3 Waste reduction audit

The five high-impact changes selected for evaluation were: change from disposable to re-useable skin prep applications; re-useable drapes; change of the probe used for making the lead tract; rationalisation of the instrument sets; and adoption of the ‘The Green Surgery’ checklist. After each DBS operation, waste was categorised for disposal according to standard hospital procedure (comprising clinical, domestic, recycled, and re-useable) and weighed with a calibrated portable digital hanging scale. An emission factor of 0.64, e.g. 10 kg of waste = 6.4kgCO2e was obtained from the UK Government's 2024 greenhouse gas emission conversion factors, published by the Department for Energy Security and Net Zero (Department for Energy Security and Net Zero).

### 2.4 Product Carbon Footprint (PCF)

This evaluates the total greenhouse-gas emissions involved in the manufacture of any one specific component throughout its life cycle. This includes raw-material extraction, manufacturing, packaging, transportation, utilisation, and disposal. We sought to perform this on the probe used for making the lead tract as there were three options all of which appeared equivalent. These were: Boston Scientific Stereotactic TCD Electrode (Boston Scientific Corporation, Malborough, Mass., USA); Inomed triple-lumen biopsy needle (inomed Medizintechnik GmbH, Emmendingen, Germany); and Nashold Biopsy Needle (IntegraLife, Princeton, NJ, USA). A similar method was used to evaluate consumable use involved in the implementation of our five high-impact changes. Consumables were quantified based on weight of material and manufacturing process. Where weight was not available, it was calculated from reverse engineering images in 3D CAD software. Data was reported as carbon dioxide equivalents (CO2e) which accounts for different greenhouse gases.

### 2.5 Statistics

Estimation of effect size was hindered by the lack of evidence available to demonstrate the effectiveness of individual interventions. Practically, the lead surgeon performed approximately twenty new full DBS operations per year. We wished to complete the audit loop within a year, therefore our initial plan was to compare ten in each arm, which would allow us an 80% power to detect a 30% change in effect size. However, during the first part of the audit, it became apparent that due to the CE-marking expiring on the stereotactic frame consumables, we would have to stop performing surgery and therefore we curtailed our audit plan to a consecutive series, n = 16 (8 in each arm).

All sample sizes are presented as mean (M) and standard deviation (SD). Comparisons were performed with independent two-sided Student's t-tests. Statistical significance was set at p < .05.

## 3 Results

### 3.1 Clinical data

**Supplementary Fig. 1:**
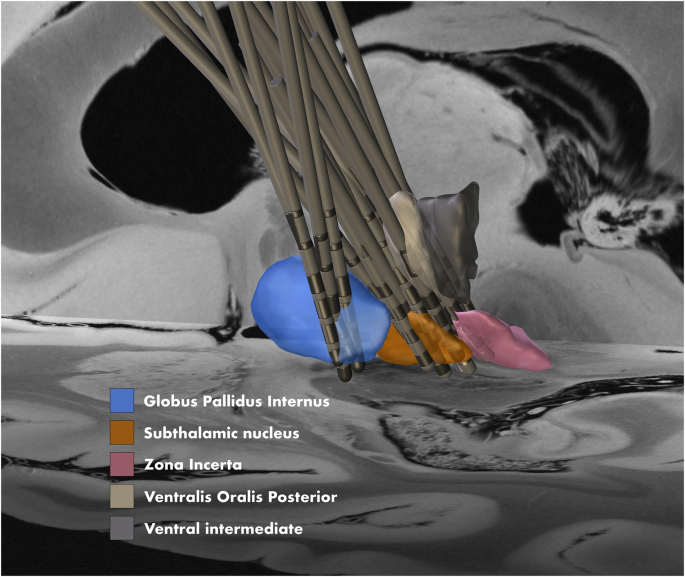
**Lead Localisation** All 16 cases have had their leads localised with Lead-DBS (www.lead-dbs.org) (Neudorfer et al., 2023). Leads have been flipped to the left hemisphere and viewed at a 45° oblique angle to allow visualisation of all targets used. Typical targets include: globus pallidus internus; subthalamic nucleus (STN); dual-targeted ventralis oralis posterior (Vop) and STN; and dual-targeted VOP and zona incerta (ZI).

Between February 2024 and January 2025, we performed 16 full DBS surgeries under the care of the senior author (MGH). This included 8 prior to implementation of the 5 high-impact changes, and 8 afterwards. During the audit period, no complications were noted. Specifically, no infections occurred (*vis-a-vis* change to skin preparation and draping), while accuracy (Supplementary Fig. 1) remained at 0.34 mm (0.37 SD) (*vis-a-vis* change in DBS probes) according to pre-defined methodology based on distance of closest electrode to the corresponding atlas target (Hart et al., 2022). Operative time (from knife-to-skin to sign-out, including transfer to CT) was 131 min (15.5 SD) with no difference between arms.

### 3.2 Waste reduction

**Fig. 2:**
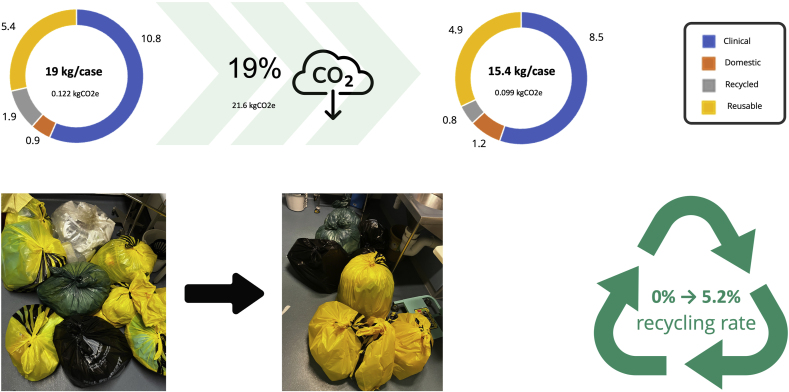
**Waste Audit** Summary of the changes in waste following the introduction of the 5 high-impact changes. Cumulatively, waste was reduced from 19 kg per case to 15.5 kg per case, resulting in a 19% reduction in CO2e. Effective recycling was 0% at baseline due to logistical and communication errors in defining what waste was suitable for recycling. Following review and communication of guidelines, recycling was 5% of total waste. No changes were statistically significant before and after the audit.

At baseline, 19 kg (SD) of waste was generated per DBS implantation, resulting in 12.16 kgCO2e (Fig. 2). This was broken down as 10.8 kg (57%) clinical, 5.4 kg (28%) re-useable, 0.9 kg (5%) domestic, and 1.9 kg (10%) recycling. Following implantation of the 5 high-impact changes, waste was 15.5 kg per case, resulting in 9.92 kgCO2e. This was broken down as 8.5 kg (55%) clinical, 4.9 kg (32%) re-useable, 1.2 kg (8%) domestic, and 0.8 kg (5%) recycling.

**Supplementary Fig. 2:**
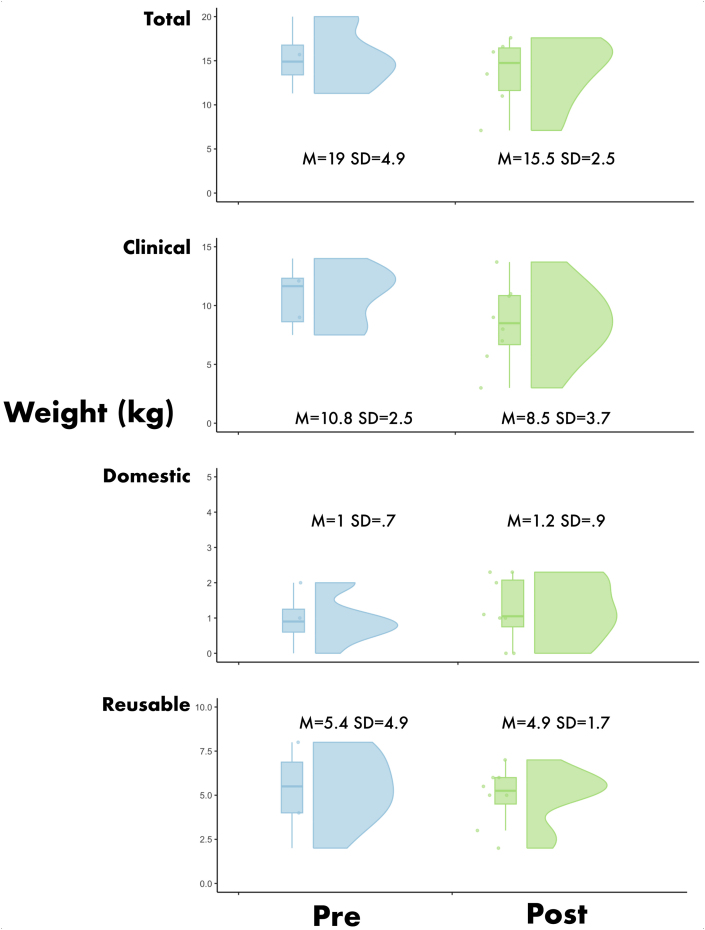
**Waste Management Audit** Summary of the changes in waste following the introduction of the 5 high-impact changes. No changes were statistically significant before and after the audit.

Total waste before (M = 19.0, SD = 4.9) and after (M = 15.5, SD = 4.7) was no different (*t* (Zygourakis et al., 2017) = 1.49, *p* = .16). There was no change in clinical (Pre M = 10.8 SD = 2.5, Post M = 8.5 SD = 3.7, *t* (Zygourakis et al., 2017) = 1.54, *p* = .15), re-useable waste (Pre M = 5.4 SD = 4.9, Post M = 4.9 SD = 1.65, *t* (Zygourakis et al., 2017) = 0.53, *p* = .60), or domestic (Pre M = 1 SD = 0.7, Post M = 1.2 SD = 0.9, *t* (Zygourakis et al., 2017) = -0.52, *p* = .61) (Supplementary Figure 2).

During the first phase of the audit, it became apparent that there was a discrepancy between the hospital instructions for determining if waste was recyclable or not, and those defined in waste management. Specifically, inclusion of any plastic within waste marked for recycling was deemed to be unrecyclable, even though an estimated two-third of operating theatre plastics are considered potentially recyclable (McGain et al., 2015). This was checked every 5th bag (rather than every bag), and if the contents within were found to be in breach of the waste management and recycling policy, it led to exclusion of not only this bag but the next 5 bags from recycling. This process was then repeated, as is standard practice for recycling (at least in the UK). The upshot of this discrepancy between hospital and waste management guidance was that despite recycling 1.9 kg (10%) of waste at baseline, the actual recycling rate was 0%. Subsequently, this increased to 5%.

### 3.3 High-level Product Carbon Footprint (PCF)

**Fig. 3:**
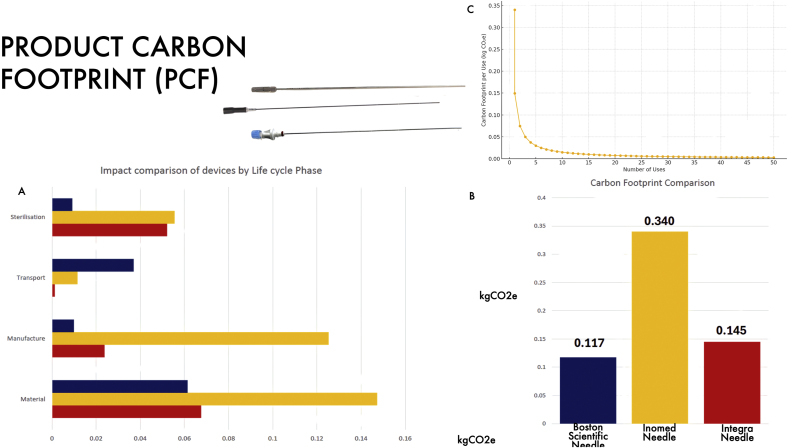
**Product Carbon Footprint** A: comparison of devices according to different life cycle phases. B: Overall kgCO2e for each of the three needles. C: Demonstration of the kgCO2e for the re-useable Inomed Needle over time and number of uses. By three uses it has equivalent kgCO2e as the disposable needles.

**Supplementary Fig. 3:**
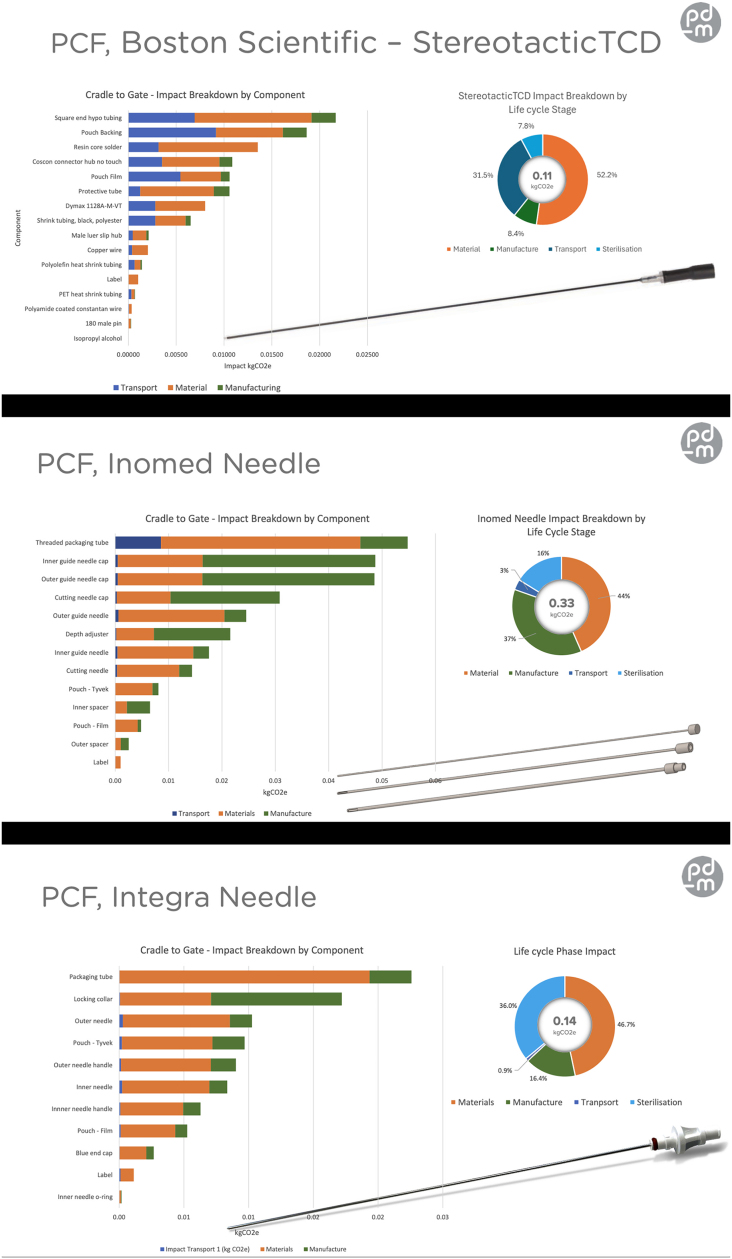
**Individual Product Carbon Footprints** Detailed product carbon footprints for each of the three devices (Boston Scientific Needle, Integra Needle, Inomed Needle). Cradle-to-grade breakdown per component, as well as total kgCO2e breakdown according to life-cycle stage.

Next, we performed a high-level product carbon footprint (PCF) analysis of three commonly available probes used for making the tract with which we placed our DBS leads. This is a critical part of our procedure in terms of accuracy, safety, and infection. It was also anticipated that this was one of the most costly changes, but which had a potentially high (albeit unknown) impact on carbon utilisation. Our analysis (Fig. 3) found that the lowest kgCO2e was for the Boston Scientific Needle (0.11kgCO2e), closely followed by the Integra Needle (0.14kgCO2e), and finally the Inomed Needle (0.33kgCO2e). However, as the latter was re-useable, it would be expected to make net reductions in kgCO2e after only three uses. Specifically, manufacturing and materials were significantly higher for the re-useable Inomed Needle, as expected given it is designed to be re-useable. The Boston Scientific Needle had the lowest use for sterilisation but the highest use for transportation. A detailed analysis of all individual components categorised as materials, manufacturing, transportation, and sterilisation is presented in Supplementary Figure 3.

### 3.4 Consumable review

**Fig. 4:**
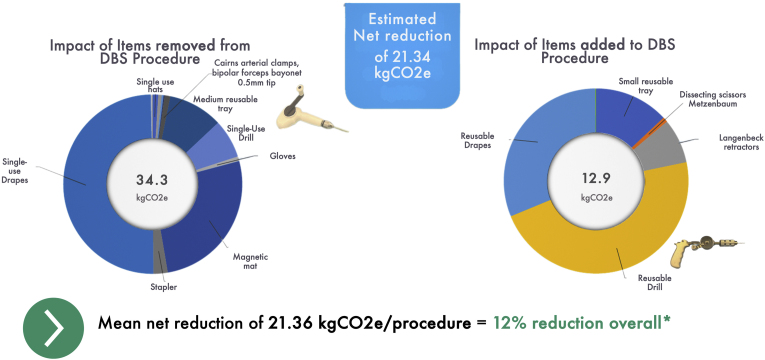
**Consumable Reduction** Impact of changes in consumables used in our DBS procedure at a single-case level. On the left, a summary of items removed from our DBS procedure in order to reduce the carbon footprint. Over half the reduction was in removing single-use drapes, whereas other changes to our sets and ‘pick lists’ made up the remainder of the savings. On the right is a summary of the impact of items added to the DBS procedure. Re-useable drapes had a major impact but still resulted in a net reduction per case. Replacement of a single-use versus re-useable drill had a net increase at a single-case level but over time it would produce a net reduction. ∗Average total of 173kgCO2e/operation (MacNeill et al., 2017).

Finally, we performed a similar PCF analysis to understand consumable use per DBS procedure (Fig. 4). Specifically, we sought to estimated kgCO2e saved in removing items from our procedure, versus any potential gain in having to add in replacement items. In total, we removed items of 34.4 kg CO2e. The majority of this was with single-use drapes (17 kg CO2e or 50%) which weighed approximately 1 kg int total. Changes to our pick lists resulted in the next highest reduction (30%) followed by changes to the components in our sets (13%).

However, items were required to be added, for example as replacements, which came to 12.9kgCO2e. Of these, the highest addition was in adding re-useable drapes (47%), however the net impact in kgCO2e was in favour of this change (a reduction from 17.1kgCO2e to 4.0kgCO2e). The next highest gain was in replacing the disposable drill (2.4kgCO2e) with a re-useable drill (6.1kgCO2e), however this would clearly work in favour after only 3 DBS procedures. Overall, we removed 17 items and added in 6 items, for a net saving of 21.36kgCO2e or 12% of the total use of the DBS procedure (Fig. 4).

## 4 Discussion

Implementing our 5 high-impact changes for greener DBS surgery resulted in a net saving of 21.46kgCO2e or 12% of the total kgCO2e used in the case. Over the course of the one year and twenty DBS procedures, we would save 42.96kgCO2e per case, equivalent to 859kgCO2e per year, or driving 3191 miles (approximately the distance from our hospital in South West London to Timbuktu). There was no statistically significant change in either total waste or the individual waste categories. However, our work did identify a communication error in the definition of recycled waste, allowing this to improve from 0% to 5%. Finally, a deep-dive product carbon footprint analysis of the DBS probe found that while the re-useable Inomed Needle had the highest associated kgCO2e, its reusability meant that there was a net gain after only three cases.

**Supplementary Fig. 4:**
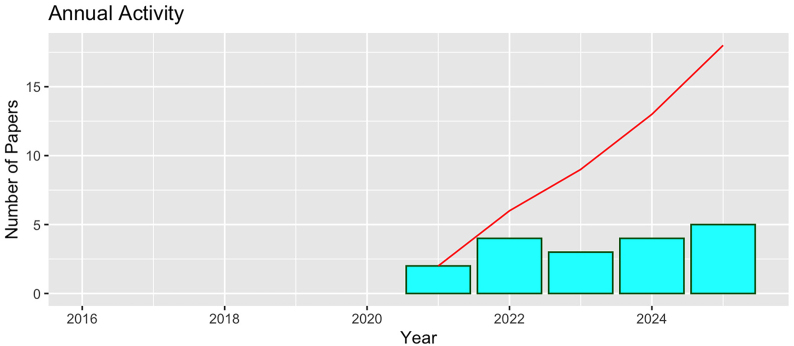
**Research on NetZero Surgery** A PubMed search was performed on “netzero” and “surgery” to identify papers over the past decade. In total, 18 papers were identified, of which 1 related to neurosurgery.

Few studies have been performed in making surgery greener (Supplementary Figure 4), and only one study has attempted this in neurosurgery (Hodnett et al., 2024). In this study a ‘Green Operating Day’ was run and ten cases included, resulting in reducing carbon emissions by 31%, or 1.04 tonnes CO2e. Reasons for the lack of studies in the field may be due to a lack of scientific interest, generalised acceptance and adoption of available measures (e.g. Green Surgery Checklist) without further appraisal, or a publication bias in that changes implemented did not have the desired effect or that applicable study designs are not commonly agreed upon. On the contrary, we would argue that all of the above are genuine reasons to spur on research into making surgery greener. Adoption of any change to surgical procedures is complicated and should be thoroughly appraised to ensure that they have the intended benefits without adverse consequences. Current study designs do not appear to be readily applicable to the field which will require innovation and refinement to properly test or refute hypotheses. Deep-dive appraisals of surgical procedures are inherently valuable in driving innovation and obviating redundant or ineffective practice. Finally, climate science has proven itself to be a rapidly growing, high-profile, multi-disciplinary, and fertile scientific niche that offers abundant opportunities for translational research within medicine, surgery, and neurosurgery (Luers, 2025).

It is disappointing that our attempts to reduce our kgCO2e use did not result in any statistically significant changes in the total amount of waste or its separate categories. It would be reassuring to believe that our procedure was already relatively optimised in terms of being green and therefore have little room for improvement. However, alternative explanations include a lack of statistical power, insensitivity of the analysis approach, and difficulties in proving benefits at an individual surgeon level. One high-impact change that we wanted to make was to introduce intraoperative CT, which would reduce transfer times and consumables used therein, as well as improving theatre utilisation (which is the highest kgCO2e drain by far). However, while we have two intraoperative CT scanners available in our department, our theatres are currently not lead-lined nor is there funding available to do so, hence they are not able to be used. This highlights the significance of logistical barriers involved and how real-world decisions are often compromises.

Limitations of our work include the relatively low numbers. However, we were constrained by wanting to provide a homogenous sample under a single surgeon over a one-year period. Therefore, our findings should be treated as preliminary until replicated by other groups. Indeed, it will be interesting if other centres with different workflows can demonstrate similar improvements. We limited our analysis specifically to the DBS procedure itself to make the project tractable, but the whole pathway for DBS is long and involves multiple hospital visits which may have a more significant effect on kgCO2e in an individual patient's journey. Additionally, parts of our methodology were determined by waste categorisation (i.e. recyclable, clinical, domestic) which likely vary between centres and therefore may also affect results (for example by having a wider range of materials open to recycling). One component of surgery that we have not sought to analyse is the kgCO2e related to the devices themselves, which is a complex feature of hardware manufacturing, packaging, and longevity (with potential savings related to rechargeable batteries depending on time utilised). However, we would hope that the attention from work such as this will spur manufacturers on to optimising their practices and making these data publicly available. Finally, significant components of carbon use, such as where a hospital sources its power from and insensible losses of energy from estates, are out of individual clinician's control and will require political lobbying to instigate meaningful changes.

In the future, having demonstrated the feasibility of our approach, we would like to expand this approach to “NetZero-DBS” across all our surgeons and indeed all neurosurgical procedures, which we hope will allow further real-world validity to our conclusions as well as increasing statistical power. Changes to our service that may help include lead-lining our theatres so we can use our intraoperative CT, fixing our automatic taps for scrubbing, using absorbable beads to contain wash allowing it to be flushed down the sluice (rather than being included in waste bags), and improving the range of materials that our recycling department can manage. We hope that our “NetZero-DBS” framework (Table 1), rather than being a universal panacea, will be an inspiration for other departments to adopt a similar methodology for appraising their DBS procedure and making changes to make surgery greener.

**Table 1:**
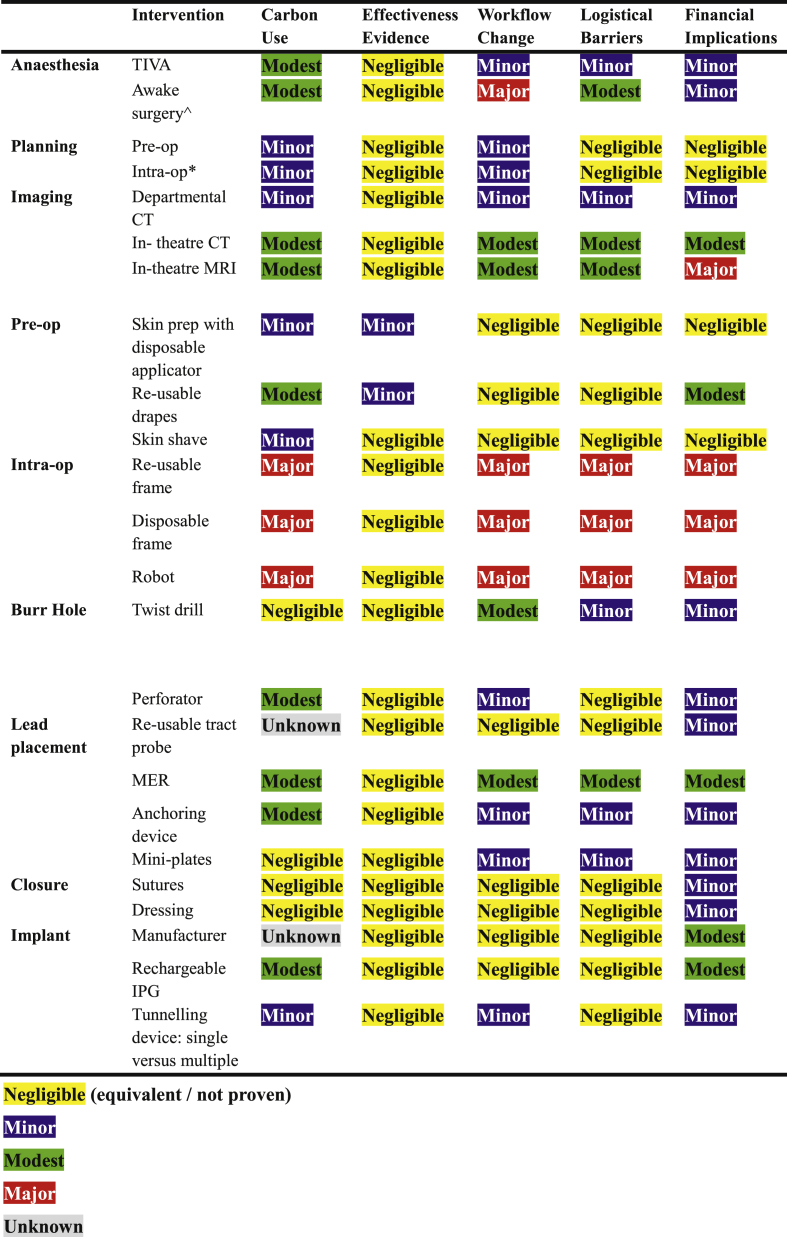
NetZero DBS Checklist Individual steps during DBS implantation surgery are broken down into their various permutations or choices in how they are carried out (e.g. inhalational or TIVA anaesthesia, awake or asleep surgery, etc). Factors are weighted according to their carbon use, evidence of efficacy, workflow changes, logistical barriers, and financial implications. Weightings were given as unknown if there was no evidence available, negligible if deemed to be equivalent or no proven difference, through to major e.g. expected seven-figure capital spend etc. Given the paucity of the literature this table is not meant to be definitive. Rather, it is designed to be a template for individual centres to adopt as appropriate, and in this case is specific to our centre and decision making for our pathway. ^Initial awake/sedation (e.g. MER, macrostimulation) followed by GA for IPG implantation. ∗vis-a-vis time under anaesthesia while planning CT: computed tomography, GA: general anaesthesia; IPG: implantable pulse generator, MRI: magnetic resonance imaging, TIVA: total intravenous anaesthesia.

## 5 Conclusions

We present an ambitious “NetZero-DBS” approach and pilot study to making a specialist neurosurgical procedure greener. Specifically, we have proven the utility of this approach for rationalising decision making for individual steps in the procedure, and a multimodal framework for appraising the impact of these changes including waste audit, product carbon footprints, and consumable use. Nevertheless, evidence of the benefit of these changes at an individual surgeon level remained elusive, cautioning against the blind-adoption of apparently obvious ‘green’ changes, and suggesting that meaningful changes to making surgery greener will require political solutions.

## Consent for publication

Not applicable.

## Author contributions

MH conceptualised the study and drafted the manuscript. TS designed the methodology and performed the formal analysis. All authors provided critical review and approved the final version.

## Statements and declarations

Nil.

## Ethical considerations

Approval was provided by St George's NHS Foundation Trust as a Review of Service.

## Consent to participate

Not applicable.

## Funding

The Product Carbon Footprint was performed by Pd-m International Ltd, funded by Boston Scientific.

## Declaration of competing interests

The authors declare no competing interests.

## Data Availability

The datasets analysed during the current study are available from the corresponding author on reasonable request.

## References

[bib1] UHAoC Change, Brighton, Sussex Medical School CfSH (2023).

[bib2] Department for Energy Security and Net Zero Greenhouse gas reporting: conversion factors 2024 2024. https://www.gov.uk/government/publications/greenhouse-gas-reporting-conversion-factors-2024.

[bib3] Hart M., Posa M., Buttery P., Morris R. (2022). Increased variance in second electrode accuracy during deep brain stimulation and its relationship to pneumocephalus, brain shift, and clinical outcomes: a retrospective cohort study. Brain and Spine.

[bib4] Hodnett R., Murphy M., Williams A., Slator N., Love-Jones S., Wigfield C. (2024). Towards net-zero operating in neurosurgery. Br. J. Neurosurg..

[bib5] Luers A. (2025). Net zero needs AI—Five actions to realize its promise. Nature.

[bib6] MacNeill A.J., Lillywhite R., Brown C.J. (2017). The impact of surgery on global climate: a carbon footprinting study of operating theatres in three health systems. Lancet Planet. Health.

[bib7] McGain F., Jarosz K.M., Nguyen M.N.H.H., Bates S., O'Shea C.J. (2015). Auditing operating room recycling: a management case report. A and A Practice.

[bib8] Mostofi A., Baig F., Bourlogiannis F., Uberti M., Morgante F., Pereira E.A. (2021). Postoperative externalization of deep brain stimulation leads does not increase infection risk. Neuromodulation: Technol. Neural Interface.

[bib9] Neudorfer C., Butenko K., Oxenford S., Rajamani N., Achtzehn J., Goede L. (2023). Lead-DBS v3. 0: mapping deep brain stimulation effects to local anatomy and global networks. Neuroimage.

[bib10] NHS England (2022). https://www.england.nhs.uk/greenernhs/wp-content/uploads/sites/51/2022/07/B1728-delivering-a-net-zero-nhs-july-2022.pdf.

[bib11] Rizan C., Steinbach I., Nicholson R., Lillywhite R., Reed M., Bhutta M.F. (2020). The carbon footprint of surgical operations: a systematic review. Ann. Surg..

[bib12] Robb H.D., Pegna V. (2023). The intercollegiate green theatre checklist. Bull. Roy. Coll. Surg. Engl..

[bib13] Rodríguez‐Jiménez L., Romero‐Martín M., Spruell T., Steley Z., Gómez‐Salgado J. (2023). The carbon footprint of healthcare settings: a systematic review. J. Adv. Nurs..

[bib14] Talibi S.S., Scott T., Hussain R.A. (2022). The environmental footprint of neurosurgery operations: an assessment of waste streams and the carbon footprint. Int. J. Environ. Res. Publ. Health.

[bib15] Zygourakis C.C., Yoon S., Valencia V., Boscardin C., Moriates C., Gonzales R. (2017). Operating room waste: disposable supply utilization in neurosurgical procedures. J. Neurosurg..

